# Chemical Composition, Antibacterial and Antifungal Activity of the Essential Oil from *Cistus ladanifer* L.

**DOI:** 10.3390/plants10102068

**Published:** 2021-09-30

**Authors:** Jamila El Karkouri, Mohamed Bouhrim, Omkulthom Mohamed Al Kamaly, Hamza Mechchate, Amal Kchibale, Imad Adadi, Sanae Amine, Souâd Alaoui Ismaili, Touriya Zair

**Affiliations:** 1 Research Team of Chemistry Bioactive Molecules and the Environment, Laboratoire des Matériaux Innovants et Biothenologie des Ressources Naturelles, Faculty of Sciences, University Moulay Ismaïl of Meknes, BP 11201, Zitoune, Meknes 50003, Morocco; jamilakarkouri@yahoo.fr (J.E.K.); mohamed.bouhrim@gmail.com (M.B.); amal_cer@yahoo.fr (A.K.); imadadadi@gmail.com (I.A.); amina.sanae2020@gmail.com (S.A.); alaouiismaili1@gmail.com (S.A.I.); 2 Department of Pharmaceutical Sciences, College of Pharmacy, Princess Nourah Bint Abdulrahman University, Riyadh 11564, Saudi Arabia; omalkmali@pnu.edu.sa; 3 Laboratory of Biotechnology, Environment, Agri-Food, and Health, Faculty of Sciences Dhar El Mahraz, University Sidi Mohamed Ben Abdellah, P.O. Box 1796, Fez 30000, Morocco

**Keywords:** *C. ladanifer* var. *maculatus* Dun, essential oil, antimicrobial activity, yeast, mold, chemical analysis

## Abstract

*Cistus ladanifer* L. is a plant widely used in folk medicine to treat various illnesses. This study aims to evaluate the effect of the plant flourishing time harvest on the chemical composition and the antimicrobial effect of its essential oil. Chemical analysis of the essential oil was carried out using gas chromatography-mass spectrometry (GC-MS). The antibacterial and antifungal proprieties were tested against four selected bacteria (*Staphylococcus aureus*, *Salmonella Typhi*, *Escherichia coli*, and *Acinetobacter baumannii*) and nine fungi (Yeasts (*Candida tropicalis*, *Candida glabrata*, *Candida dubliniensis*, *Candida* sp., *Rhodotorula rubra*, *Cryptococcus neoformans*) and molds (*Penicillium* sp. (P), *Fusarium* sp. (F), *Aspergillus niger* (*A. niger*)), respectively. The essential oil of *C. ladanifer* demonstrated a powerful antibacterial activity with an inhibition zone of 55 ± 0.22 mm *for Staphylococcus aureus,* 42 ± 0.11 mm for *Escherichia coli,* 35 ± 0.27 mm for *Acinetobacter baumannii (Full resistant to antibiotics)* and 30 ± 0.25 mm for *Salmonella Typhi.* It also inhibited all tested bacteria at 10 µL/mL. For the antifungal activity test, *C. tropicalis* and *C. neoformans* appeared to be the most sensitive strains to the essential oil with an inhibition zone of 13 mm, followed by *R. rubra* and *Penicillium* sp. (12 mm), then *C. dubliniensis* and *C. glabrata* (11 mm). The chemical analysis of the essential oil by GC-MS revealed that the major components of the essential oil were viridiflorol (17.64%), pinocarveol (11.02%), bornylacetate (9.38%), and ledol (8.85%). *C. ladanifer* exhibited a remarkable antimicrobial activity that could be more exploited to develop targeted natural remedies against specific diseases.

## 1. Introduction

Aromatic and medicinal plants (AMPs) have long been a part of man’s everyday existence for a variety of purposes. AMPs play a crucial and fundamental role in traditional medicine and play a very significant role in drug discovery [1]. Natural substances account for around 25% of all medicines available for the treatment of illnesses (plants, animals, bacteria, and fungi) [2]. *Cistus ladanifer* L. is one of the medicinal plants, belonging to the Cistaceae family, the latter represented by seven genera (*Cistus, Fumane, Halimium, Tuberaria, Helianthemum, Hudsonia* and *Lechea*), the genus *Cistus* alone encompass 16 species particularly distributed in the Mediterranean region [3,4], this kind is widespread in Portugal, Spain, Italy, Algeria, and Morocco [5]. The most common species are *C. ladanifer* (Gum Cistus), *C. monspeliensis* (Montpellier Cistus), *C. salviifolius* (Sage Cistus), *C. laurifolius* (Laurel Cistus), *C. creticus* (Cretan Cistus), and *C. albidus* (Cottony Cistus). *C. ladanifer* is represented in Morocco by two varieties that differ mainly by the color of the petals of flowers: *C. ladanifer* var. *albiflorus* Dun with completely white petals and *C. ladanifer* var. *maculatus* Dun with petals spotted with crimson. *C. ladanifer* is a much-exploited plant in Morocco, it is known as ‘Touzzalt’ in Amazigh, and in Arabic, it is called kastousse, Bouzegzaw, ftah, Targla, Touzzala’ [6]. It is a very fragrant spontaneous shrub; it has sticky branches up to 2 m tall, large white flowers (64 mm in diameter) that appear during spring (March–May) and have a three-day lifespan. Its seeds appear between July and October [7]. This species grows in very diverse climates; it is extremely resistant to cold stress, drought, and high temperatures [8].

In Oulmes (Middle Atlas, Morocco), the local population uses *C. ladanifer* traditionally to treat various diseases and health issues due to its antioxidant, gastric, anti-inflammatory, antitumor, antimicrobial, and antiviral properties [9,10,11]. This plant is usually harvested in May in this area (flowering time) [12]. The leaves of all *Cistus* species secrete essential oils [13]. Essential oils from different species of medicinal plants have been documented to possess antimicrobial propriety with strong activity against Gram-negative and Gram-positive bacteria and also fungi [14].

*C. ladanifer* essential oil is characterized by a large number of sesquiterpenes (viridiflorol and ledol) and monoterpenoids (bornyl acetate and pinocarveol) [15] that are maybe behind several reported activities such as analgesic, anti-inflammatory [16], antiplatelet [17], antioxidant [10,18], antidiarrheal, antispasmodic, anti-acid [19,20], antiulcer [21], antitumor and gastroprotective activities [22]. Sosa et al. confirmed that aqueous *Cistus* extract could be considered an inhibitor of calcium transport in skeletal muscles [23]. Belmoukhtar et al. and Aziz et al. showed that aqueous extract of *C. Ladanifer* has also been considered as a curative and preventive treatment for hypertension and the possibility of using it in the treatment of gastrointestinal disorders [20,24]. Amensour et al. reported that *C. ladanifer* is a source of natural antioxidants that can be exploited in the food industry given the high levels of existing flavonoids and phenolic compounds [25]. Finally, Andrade et al. and Barrajón-Catalánet al. have also reported its cytotoxic potential against several human cancer cells [10,11]. Due to bacterial and fungal resistance and the side effects of antibiotics, great interest has been given to biologically active molecules isolated from plant species [26], in particular, essential oils [19]. The objective of this work is to study for the first time the effect of *C. ladanifer* essential oil at its flourishing time (April) and the collection region (Oulmès region, Middle Atlas) on the chemical composition and antimicrobial effect of *C. ladanifer* var. maculatus Dun.

## 2. Results

### 2.1. C. Ladanifer Moisture Content and Its Essential Oil Yield Percentage

The moisture content of *C. ladanifer* essential oil was 13.2%. The yield extraction of the essential oil was around 0.21 ± 0.01%. These results were quite high compared to those obtained by several other researchers. In France, Robles reported a yield of 0.119 ± 0.016% [27]. In Algeria, Bechlaghem reported a yield of 0.08% [28]. Zidane et al. from Morocco obtained a yield of 0.14% [29]. Grech et al., also from Morocco found a yield of 0.3 to 0.4% in the northern region [30]. Thus, we find that the *C. ladanifer* essential oil yield, although highly variable, remains relatively low regardless of the region and the time of harvest.

### 2.2. Mineral (ash) and Organic Matter Content in the C. Ladanifer Essential Oil

The mineral content obtained was 3.7%, which was considered quite important. The organic content was around 1.35%. This variation can be explained by the mineral reserves of the soil, the efficiency of their root capture, and their movement towards the aerial organs of the *C. ladanifer*.

### 2.3. Refractive Index and Brix Index of the C. Ladanifer Essential Oil

The refractive index and Brix index are qualitative identification characteristics that may be used to evaluate the purity of essential oils [31]. Each substance has its specific refractive index. The purity of a product is determined by how near its refractive index is to the anticipated value. The refractive index of our studied essential oil is 1.45. Mrabet et al. reported a refractive index of 1.49 for the essential oil of *C. ladanifer* var maculatus from northern Morocco [32]. The refractive index values of *C. ladanifer* essential oil extracted by hydrodistillation are comparable to those of standards, indicating that our extracts are of excellent purity confirmed also with the low Brix index (1.33) which is an indicator of the concentration (%) of all solids dissolved in the essential oil (Table 1).

### 2.4. GC-SM Analysis of the Essential Oil of C. Ladanifer

**Figure 1:**
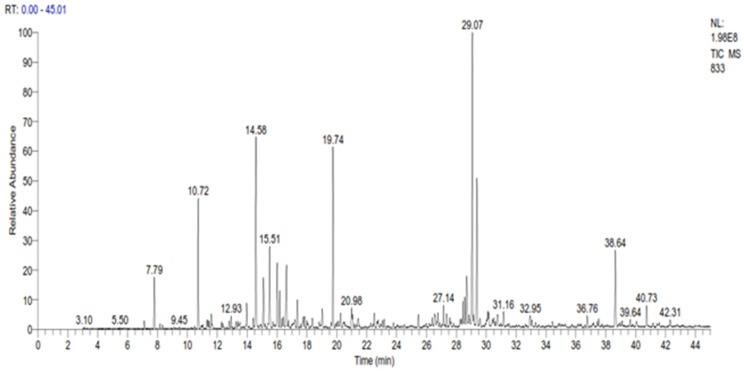
GC Chromatogram of the essential oil of *C*. *ladanifer*.

The GC-MS analysis revealed the presence of 35 compounds in the essential oil of *C. ladanifer* (Figure 1). These compounds were divided into oxygenated sesquiterpenes (34.02%), oxygenated monoterpenes (33.14%), linear esters (10.38%), monoterpenes (9.11%), and sesquiterpenes (4.29%). The major constituents present in this EO were viridiflorol (17.64%), *trans*-pinocarveol (11.02%), bornylacetate (9.38%), and ledol (8.85%) (Table 2). The percentage of these constituents is higher than those found by Boukil et al. (oxygenated hydrocarbons (13.27%), oxygenated sesquiterpenes (2.57%), and monoterpenic ester (5.86%)) [19]. The same authors found that the main components from the fresh leaves of *C. ladanifer* were verticiol (18.16%), camphene (17.70%), *n*-butylcyclohexane (5.95%), and 3-carene (5.23%) [19]. These findings indicate that the time of harvest has a significant impact on the chemical composition obtained. The results of various chemical analysis studies on *C. ladanifer* essential oil carried out previously showed that the main constituents of *C. ladanifer* leaf essential oil from Northern Morocco were viridiflorol (19.6%), bornyl acetate (16.7%), and camphene (12.3%) [30]. Zidane et al. characterized *C. ladanifer* from Eastern Morocco and indicated the presence of camphene (15.5%), borneol (11.1%), 2,2,6-trimethylcyclohexanol (7.3%), 4-terpineol (6.3%), and α-pinene (4.2%) as the major compounds in the essential oil of this plant [29]. In Algeria, it was found that the main constituents of this oil were 5,7-di-*epi*-α-eudesmol (13.6%), borneol (12.5%), camphene (12.2%), δ-cadinene (7.6%), α-eudesmol (6.4%)%), 4-terpineol (5.7%) and α-pinene (4.2%) [28]. In France, Verdeguer et al. characterized the chemical composition of the oil extracted from the leaves and stems of *C. ladanifer* of Spanish origin but cultivated in Corsica by the presence of pinene (39%), viridiflorol (11.8%), ledol (3.3%) and bornyl acetate (3.1%) [33]. In Portugal, the chemical composition of *C. ladanifer* oil shows the presence of three sesquiterpenes alcohols, viridiflorol (13.6–17.4%), globulol (3.1–5.0%), and an unknown alcohol sesquiterpene (2.7–6.0%), as well as diterpene alcohol 15-*nor*-labdan-8-ol (1.7–5.2%) [34]. In Spain, the composition of the essential oil of *C. ladanifer* cultivated in central Spain, revealed its richness in oxygenated compounds, with *trans*-pinocarveol (20.00%), bornyl acetate (7.03%), and terpinen-4-ol (6.37%) as the main monoterpene compounds. Viridiflorol (13.59%) and ledol (4.36%) were the main constituents of the oxygenated sesquiterpene fraction. Large amounts of α-pinene (4.70%) were found in the hydrocarbon fractions. From this comparison, it seems that Moroccan *C. ladanifer* essential oil composition is closer to that of Corsica with Spanish origin. The chemical composition of *C. ladanifer* essential oil varies considerably depending on the source, plant material, and extraction method. As a result, based on the intended product during the exploitation of the species, a selection of organs, vegetative stage, and area proves to be extremely helpful in promoting the acquisition of very accurate chemotypes.

### 2.5. Antibacterial Activity

In this study, the antibacterial activity was evaluated using two methods: the agar disk diffusion method and the dilution in a liquid environment. The aim of the tests is to highlight the inhibitory power of the essential oil vis-à-vis the tested bacteria after 24 h of incubation at an adequate temperature of 37 °C.

#### 2.5.1. The Antibiotic Sensitivity Test

The antibiotic sensitivity profiles of the strains are developed according to the recommendations of the Committee on Antibiotic susceptibility of the French Society for Microbiology (CA-SFM) and presented in Table 3. From the table, it can be concluded that the strains of *S. aureus* and *S. Typhi* were sensitive to all antibiotics, while *E. coli* was only resistant to ticarcillin. At the same time, *A. baumannii* demonstrated complete resistance to all tested antibiotics. This may be due to the higher resistance of Gram-negative bacteria due to the complexity of their cell wall, containing a double membrane in opposition to the single glycoprotein/teichoic acid membrane of Gram-positive bacteria [35]

#### 2.5.2. The Agar Disk-Diffusion Method for *C. ladanifer* Essential Oil

**Figure 2:**
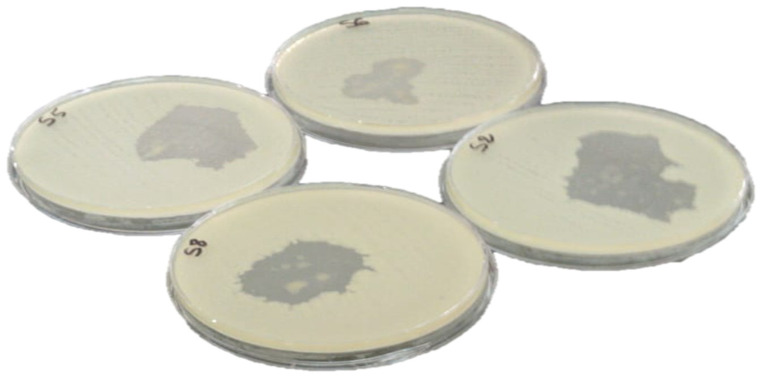
Bacterial strain growth inhibition zone of *C. ladanifer* essential oil.

Table 4 and Figure 2 represent the results of the agar disk-diffusion method for *C. ladanifer* essential oil against the selected bacterial strains after 24 h at 37 °C. The agar disk diffusion method results indicated that the essential oil of *C. ladanifer* has a remarkable antibacterial activity compared to the concentration used (5 μL). From the analysis of the results obtained (Table 4), it was noted that the four microorganisms studied are sensitive except *S. Typhi* which demonstrated an inhibition zone of 30 ± 0.25 mm when the EO of *C. ladanifer* was used. The best inhibition diameter was against *S. aureus* (55 ± 0.22 mm) and *E. coli* (42 ± 0.11). It was also noted that contrary to the sensitivity test when *A. baumannii* demonstrated full resistance to the tested antibiotics, it demonstrated an inhibition diameter of 35 ± 0.27 when the EO was used, indicating a good and promising effect. Our results are superior to those found by Benayad et al. (*S. aureus* (28 mm), *A. Baumannii* (24 mm), and *E. coli* (18 mm)) (plant harvested in May) [11]. Same note for results obtained by Boukil et al. (*S. aureus* (14 mm), and *E. coli* (9 mm)) (plant harvested in August) [19]. As an outcome, harvesting the plant at the flowering stage is correlated to a potent antibacterial power.

#### 2.5.3. Minimum Inhibitory Concentrations (MIC) and Minimum Bactericidal Concentrations (MBC)

During this investigation, the determination of the MIC was evaluated by assessing the inhibitory power of the plant’s essential oil at different concentrations against the selected bacteria (Table 5). In total accordance with the previous results obtained in the agar disk diffusion method and the observed inhibition zone, the results obtained indicated that the *C. ladanifer* essential oil MIC and MBC for all strains were 10 μL/mL. Regarding the MBC/MIC activity ratio, our results indicate that it was equal to 1 for all strains. This value allows us to affirm that the essential oil of *C. ladanifer* is bactericidal. This antimicrobial activity of this essential oil can only be explained by its chemical profile rich in 34.02% of oxygenated sesquiterpenes and oxygenated monoterpenes, and 33.14% of monoterpenes which are known as versatile anti-infective agents (antibacterial and antifungal) [36]. The structures of the functional groupings of the constituents of essential oils could play a crucial role in determining the antibacterial power of essential oils [37]. Nevertheless, minority compounds can interact directly, or in a synergistic or in an antagonistic way, to create a mixture with biological activity. Guinoiseau et al. [38], Rossi et al. [39], and Vieira et al. [40] demonstrated that essential oil from *C. ladanifer* has antimicrobial activity against Gram-positive and negative pathogens of clinical importance such as *Staphylococcus aureus*, *E. coli*, *Streptococcus pneumonia*, *Pseudomonas aeruginosa*, *Enterobacter aerogenes*, and *Campylobacter jejuni*.

### 2.6. Antifungal Activity

**Figure 3:**
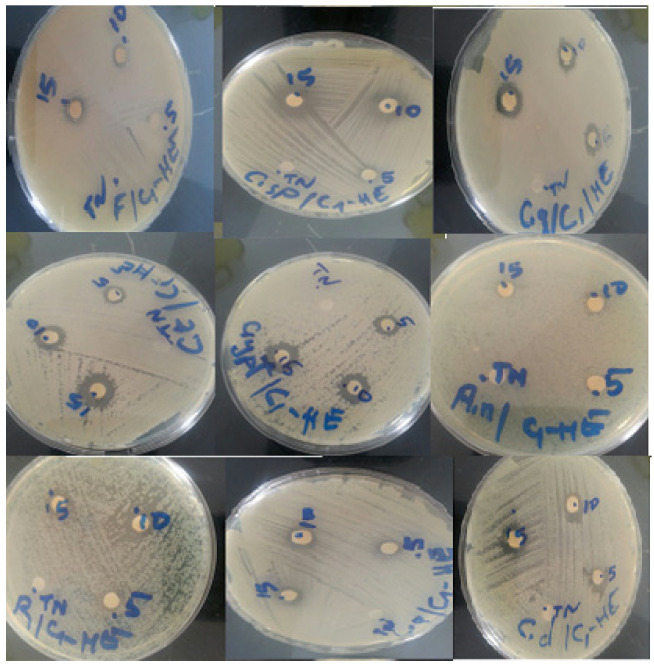
Fungal strains zone of inhibition diameters of essential oil of *C. ladanifer*.

The disc diffusion technique enabled us to demonstrate the antifungal activity of *C. ladanifer* essential oil against 10 different fungus strains (Figure 3). The antifungal effect of a volume of 5 μL, 10 μL, and 15 μL of *C. ladanifer* essential oil is presented in Table 6. The essential oil demonstrated a good inhibitory activity with a slight dose-dependent activity. Fluconazole used as a positive control presented the best inhibition zone diameters against all the selected strains. In relation to the essential oil activity, *C. tropicalis* and *C. neoformans* were the most sensitive with an inhibition zone of 13 mm, followed by *R. rubra* and *Penicillium* sp., which have the same inhibition diameter of 12 mm, then *C. dubliniensis* and *C. glabrata* with inhibition zones of 11 mm. No studies on the antifungal activity of *C. ladanifer* of our study area (Middle Atlas, Morocco) have been carried out, except for a few attempts that have been reported by Boukil et al. [16].

#### Minimum Inhibitory Concentrations (MIC) and Minimum Fungicide Concentrations (MFC)

Table 7 summarizes the values of the MIC and MFC of the essential oil determined in a liquid medium against the ten tested fungal strains. The essential oil of *C. ladanifer* showed an antifungal activity on all fungal strains tested with concentrations ranging between 16 and 64 μL/mL.

The results of the MFC were all compared to those of MIC except for *Candida* sp. strain. Those obtained results were interesting and in agreement with those obtained by Guinoiseau et al., Rossi et al., Vieira et al. which indicate that the essential oil from *C. ladanifer* has an antimicrobial activity on fungi (such as *Aspergillus niger*, *Botrytis cinerea*, *Mucorracemosus,* and *Verticilliumalboatrum*) [38,39,40].

## 3. Discussion

This study, among others, focused on revealing the potential bioactivity of the essential oil of *C. ladanifer*. The study’s novelty was that plant material was collected during its time of flowering and in a different region (Middle Atlas), which is known for its semi-arid climate. The edaphic and climate parameters and others have influenced the variation of the composition revealed in the chromatographic analysis. The essential oil major components were viridiflorol (17.64%), *trans*-pinocarveol (11.02%), bornyl acetate (9.38%), and ledol (8.85%) indicating the domination of the oxygenated sesquiterpenes (34.02%), oxygenated monoterpenes (33.14%) in the overall composition.

The oxygenated sesquiterpenes and monoterpenes are a well-known group of compounds with antibacterial and antifungal proprieties [41,42] and their presence among the EO composition explains the majority of the outstanding outcomes obtained.

The strains chosen for this research are of great interest in the areas of clinical and public health. Their increasing resistance to conventional drugs has prompted further research into new, more effective options, particularly natural products [43,44]. *S. aureus* (Gram-positive) is a member of the indigenous human microflora and may be found asymptomatically in a variety of bodily locations. Diseases caused by transmission from these locations are both endemic and epidemic [45]. Infection with *S. aureus* is a leading cause of skin, soft tissue, respiratory, bone, joint, and endovascular diseases. Many strains of *S. aureus* are becoming resistant to current antibacterial treatments, posing a significant issue in medical microbiology [46].

*S. Typhi*, one of the representatives of the *Salmonella* family, is the direct causative organism of typhoid fever [47] (accompanied by weakness, headaches, mild vomiting, abdominal pain, and constipation). Symptoms may persist for weeks or months if not treated [48].

*E. coli* usually colonizes human babies’ gastrointestinal tracts within a few hours after birth. *E. coli* and its human host often live in excellent health and mutual benefit for decades [48]. These commensal *E. coli* strains seldom cause illness unless the host is immunocompromised or the usual gastrointestinal barriers are broken, as in peritonitis. Diarrhea induced by *E. coli* infection is a growing issue in both the developing and developed worlds, with significant rates of death in newborn infants and animals [49]. Although most commensal representatives found in human and animal gut flora are non-pathogenic, certain strains are very dangerous.

The genus Acinetobacter, over the past 30 years, has experienced considerable taxonomic evolution. Its most prominent example, *A. baumannii*, has emerged as one of the most problematic infections for healthcare facilities worldwide [50]. *A. baumannii* strains resistant to all known antibiotics have now been discovered, indicating a sentinel occurrence that should be addressed by the worldwide health care community as soon as possible. It often attacks the most susceptible hospitalized patients, those who are severely sick and have compromised skin integrity and airway protection [51].

In terms of fungus, non-albicans candida species are increasingly being reported as both colonizers and pathogens causing nosocomial fungal bloodstream infections, accounting for nearly half of all non-superficial candida infections, with *C. glabrata*, *C. tropicalis*, and *C. dubliniensis* being the most common [52].

One other life-threatening fungi exploited in this study is *C. neoformans* which is responsible for cryptococcal meningitis, the most prevalent type of cryptococcosis, often chronic and deadly if left untreated [53]. This virulence is none less than those presented by other fungi such as *Aspergillus niger*, a fungus that causes the “black mold” on certain fruits and vegetables (contaminant of food) which its consumption (as it secretes ochratoxins –mycotoxins) causes nephrotoxicity and renal tumors [54]. The EO of *C. ladanifer* and through this study demonstrated a strong and real potential that could be better exploited to fight against the threats presented by all the microbial strains studied with slight differences in terms of efficacy.

## 4. Materials and Methods

### 4.1. Materials and Reagents

*Ticarcillin (TIC), ceftazidim (CAZ), Meropenem 10 (MEM10),* and *Ticarcilline85 (TIM85),* were purchased from Sigma Aldrich (St-Quentin Fallavier, France). All chemicals and solvents were of highly analytical grade and were used as received from the supplier without further purification.

### 4.2. Plant Material

*C. ladanifer* was harvested in April 2019 (time of flowering) in El Harcha forest, which is located in the province of Khemisset, circle of Oulmes, 150 km south-east of Rabat, it extends entirely over the rural commune of Teddas (Middle Atlas region with semi-arid climate). The plant material was then dried in the shade, at room temperature, and stored for various uses. Professor Mohammed Fennan, a botanical expert from Mohammed V University’s scientific institute, identified this plant where a specimen of the voucher was set under collection number RTCBME10.

### 4.3. Extraction and Chemical Analysis of C. Ladanifer Essential Oil

#### 4.3.1. Moisture Content Determination of the Plant Material

The moisture content was calculated after drying three samples (5 g) of the plant material at 100 °C in an oven until a constant weight was obtained. The calculation formula was as follows:

$$\%\mathrm{MC}=\frac{w1-w2}{w1}\times 100$$

where %MC: Moisture content of the plant material, w1: Initial mass of plant material in g introduced into the oven at the time (t_1_) and w2: Final mass of plant material in g removed from the oven at the time (t_2_).

#### 4.3.2. Essential Oil Extraction

Extraction of the essential oil was carried out by hydrodistillation using a Clevenger-type apparatus (VWR, Pennsylvania, United States). The essential oils were recovered in small opaque bottles and stored at 4 °C until further use. The yield extraction is expressed as a percentage of the volume of essential oil (mL) about the mass of the vegetable material (g). The calculation formula is as follows:

$$\mathrm{YE}=\frac{V}{w-\left(w\times \mathrm{MC}\right)}$$

where YE: Efficiency of extraction of essential oil expressed as volume of essential oil per mass of vegetable material (V/m), w: Mass of vegetable material used for oil extraction, V: Volume of essential oil collected (in mL) and MC: Moisture content of the plant material.

#### 4.3.3. Mineral (ash) and Organic Matters Content of *C. Ladanifer* Essential Oil

The method used is that described by the “Association Française de Normalisation”

(AFNOR) standard [55]. The raw ash is obtained after the destruction of the organic matter by incineration. In a desiccator, nickel crucibles were dried in the oven for 1 h and were deposited and tarnished. After cooling, each crucible was weighed at 0.1 mg. Approximately 3 g of sample was crushed at 1 mm and weighed in the crucibles at 0.1 mg. The solid crucibles are fed into the muffle furnace under the exhaust hood and their contents are burnt for 4 h at 550 °C. After this time, we let the temperature go down to 100 °C and the crucibles were taken out, cooled in a desiccator, and then weighed at 0.1 g. Total ash was calculated as follows:

$$\%\mathrm{MW}=\left(\frac{w1-w0}{\mathrm{MT}}\right)\times 100$$

where %MW: Mineral matter (ash content) expressed as a percentage of the crude product, w1: Mass of crucible containing dry residue, in grams, w0: Empty crucible mass, in grams, and MT: Mass of the test sample, in grams.

The organic matter content (OM) of the sample is the difference between the dry matter mass (DM) and the mineral matter mass expressed as a percentage:

OM=DM−MW

#### 4.3.4. Refractive Index

The refractive index of chemical compounds is important because it indicates characteristic physical properties. The refractive index of the essential oil was measured at 20 °C using a refractometer (ATAGO, Tokyo, Japan) [56].

#### 4.3.5. Brix Degree

The Brix degree gives an idea of the concentration (%) of all solids dissolved in the essential oil (sugar, salts, protein, fatty acid, etc.). Indeed, a few drops of the essential oil was deposited on the refractometer tank (ATAGO). The light beam deflection through the sample (the essential oil) was varied and indicated by a colored delimitation of the degree Brix.

#### 4.3.6. GC-SM Analysis

The chromatographic analysis was carried out on a HP 6890 series gas chromatograph (Hewlett Packard, Palo Alto, CA, USA), equipped with a DB-5 (5% phenylmethylsiloxane) capillary column (30 m × 0.25 mm × 0.25 µm film thickness), an FID detector set at 250 °C and lied by a mixture of H_2_/air gas. The injection mode is split; the carrier gas used is nitrogen with a flow rate of 1.7 mL/min. The column temperature was programmed with a rise of 4 °C/min from 50 °C to 200 °C for 5 min. The device was controlled by a HP Chemstation computer system managing the operation of the device and allowing to follow the evolution of chromatographic analyses. The GC-MS was coupled to a mass spectrometer (HP 5973 series). The fragmentation was carried out by electronic impact at 70 eV. The column used is a capillary type DB-5SM (30 m × 0.25 mm × 0.25 µm). The column temperature was programmed at a rate of 4 °C/min from 50 to 200 °C for 5 min. The carrier gas used is helium with a flow rate of 1.7 mL/min. The injection mode is split. The identification of the constituents of the essential oils studied was carried out both by the index identification method of Kovàts [57], and Adams [58], and from the mass spectral database.

### 4.4. Study of C. Ladanifer L. Antimicrobial Activity

#### 4.4.1. Microorganisms, Medium, and Antibiotics

In this study, four bacterial strains and nine fungal strains were collected from the Mohammed V University Hospital in Rabat-Morocco (Table 8). Microbial strains were seeded in petri dishes containing nutrient agar, the incubation time and temperature were 24 h and 37 °C for bacteria, 48 h and 28 °C for yeast, 7 days and 28 °C for molds to obtain well-isolated colonies. Isolated colonies were collected from young cultures obtained using an inoculator. The colonies were placed in 5 mL of sterile physiological water with NaCl (0.9%) so that the solution contained about 10 g/mL at a density ranging from 0.08 to 0.1 to 625 nm [59]. The inoculum is adjusted either by adding culture if it is too weak or sterile physiological water if it is too strong. Seeding was done directly after the preparation of the inoculum. The antibiotics used for bacterial testing were ticarcillin (TIC), ceftazidime (CEF), vancomycin (VAN), tetracycline (TET), cefalexin (CEF); colistin (COL) and amikacin (AMI).

#### 4.4.2. Diffusion Method on Agar Medium

This test was carried out on Petri dishes (90 mm) by the diffusion method in the agar media of Mueller-Hinton and Sabouraud by determining the diameter of the zone of inhibition [60,61]. Disks of 6 mm in diameter, filter paper, were cut and sterilized, then deposited delicately in the center and on the peripheries of the surface of a medium previously inoculated with the microbial inoculum (this inoculum was obtained in the form of a suspension in saline solution). Then, volumes of 5 μL of essential oil of *C. ladanifer* relating to the antibacterial tests and volumes of 5, 10 and 15 μL relating to the fungal tests were deposited on these discs. A sterile disc flooded with 6 μL of sterile physiological water was used as a negative control as well as another negative control disc impregnated with 2 μL of DMSO to check the growth of different bacterial and fungal strains. The inhibition diameters were measured around the disc after incubation in an oven, each test was repeated three times to obtain as precise an estimate as possible of the true experimental value sought.

#### 4.4.3. Liquid Dilution Method

This method was used to determine the minimum inhibitory concentration (MIC) and the minimum bactericidal (MBC) and fungicidal (MFC) concentration of the essential oil of *C. ladanifer* L. [62,63]. An amount of 2 µL of DMSO was added to the 10 tubes. Hemolysis containing the culture medium (1 mL) for each bacterial strain. Each volume of essential oil (2, 4, 6, 10, 12, 14, 16, 18, 20 and 22 µL) was introduced into a hemolysis tube. Then, a volume of 6 μL of the bacterial suspension with a concentration of 105 CFU/mL was taken and then was deposited in each of the preceding tubes and then 2 μL of DMSO was added for each microbial strain. Two sets were made, one containing the culture medium (1 mL) plus a strain (bacterial or fungal) and the other containing the culture medium (1 mL) plus 2 µL of essential oil alone. After an incubation of 24 h at 37 °C for bacteria, 48 h at 28 °C for yeasts, and 7 days for molds, the MIC of the essential oil was determined. We then inoculated the surface of a Mueller Hinton agar cast in a Petri dish with 100 μL of the contents of the tubes having a concentration greater than or equal to the MIC to determine the CMB and CMF.

## 5. Conclusions

In this study, it was demonstrated that the essential oil of *C. ladanifer* is high in oxygenated sesquiterpenes, which mediated the activities seen. The results of the antimicrobial activity showed that this essential oil was able to inhibit the growth of a wide variety of bacterial and fungal strains. The observed activity was correlated to the harvest time of this plant done in May, which influenced the phytochemical profile of the EO in comparison to its harvest in other months as seen in other studies from the literature. The essential oil demonstrated an excellent antimicrobial activity and could serve as an alternative to some medicine and antibiotics. More studies should be done to determine the toxicity and the therapeutic margin for a more controlled and oriented application.

## Figures and Tables

**Table 1:** Refractive index and Brix index of the *C. ladanifer* essential oil.

Plant	Refractive Index	Brix Index
*C. ladanifer* L. essential oil	1.45	1.33556

**Table 2:** The compounds identified in the essential oil of *C. ladanifer* after analysis by GC/MS on column DB-5.

No.	Compounds	Formulas	Percentage	IK Calculated	IK (ADAMS)
1	α-Pinene	C_10_H_16_	2.43	922	939
2	*p*-Cymene	C_10_H_14_	6.11	1004	1024
3	(*Z*)-vertocitral C	C_9_H_14_O	0.63	1028	1080
4	*p*-Cymenene	C_10_H_14_	0.57	1065	1091
5	α-Campholenal	C_10_H_16_O	1.34	1093	1126
6	*trans*-Pinocarveol	C_10_H_16_O	11.02	1110	1139
7	Pinocarvone	C_10_H_14_O	2.72	1125	1164
8	Borneol	C_10_H_18_O	4.80	1137	1169
9	Terpinen-4-ol	C_10_H_18_O	4.09	1151	1177
10	Myrtenal	C_10_H_14_O	1.76	1156	1195
11	Myrtenol	C_10_H_16_O	4.02	1168	1195
12	*trans-*Carveol	C_10_H_16_O	1.44	1189	1216
13	Carvone	C_10_H_14_O	0.54	1203	1243
14	(*Z*)-β-Damascone	C_13_H_20_O	0.97	1238	1387
15	Bornylacetate	C_12_H_20_O_2_	9.38	1259	1285
16	Carvacrol	C_10_H_14_O	0.80	1274	1084
17	Myrtenylacetate	C_12_H_18_O_2_	1.00	1296	1326
18	2,4,6-trimethoxytoluene	C_10_H_14_O_3_	0.61	1298	1483
19	(*E*)-Trimenal	C_13_H_22_O	0.77	1343	1421
20	Aromadendrene	C_15_H_24_	0.67	1438	1441
21	Viridiflorene	C_15_H_24_	0.95	1473	1496
22	2,3-Dihydro-1,1,4,5,6-pentamethyl 1*H*-indene	C_14_H_20_	0.81	1481	1522
23	*cis*-Calamenene	C_15_H_22_	1.17	1493	1529
24	δ-Cadinene	C_15_H_24_	0.68	1500	1523
25	Palustrol	C_15_H_26_O	1.14	1538	1568
26	Spathulenol	C_15_H_24_O	1.41	1542	1578
27	Caryophyllene oxide	C_15_H_24_O	2.85	1546	1583
28	Viridiflorol	C_15_H_26_O	17.74	1560	1592
29	Ledol	C_15_H_26_O	8.85	1570	1602
30	1,10-di-*epi*-Cubenol	C_15_H_26_O	0.75	1595	1619
31	Caryophylla-4 (12), 8 (13)-dien-5α-ol	C_15_H_24_O	0.64	1598	1640
32	Cadalene	C_15_H_18_	0.82	1634	1676
33	14-Hydroxy-4,5-dihydro- caryophyllene	C_15_H_26_O	0.64	1848	1706
34	Sclareol	C_20_H_36_O_2_	4.60	1924	2223
35	13-*epi*-Dolabradiene	C_20_H_32_	1.28	2013	2000
	Oxygenated sesquiterpenes				34.02
	Oxygenated monoterpenes				33.14
	Linear esters				10.38
	Monoterpenes				9.11
	Sesquiterpenes				4.29
	Others				9.06
	Total				100

**Table 3:** The test for the sensitivity of bacterial strains to certain antibiotics.

ATB	*A. baumannii*	ATB	*S. aureus*
TIC 75 μg	R	CIP 5 μg	S
CEF 30 μg	R	VAN 30 μg	S
MEM 10 μg	R	TET 30 μg	S
TIM 85 μg	R	CEF 15 μg	S
**ATB**	***E. coli***	**ATB**	***S. Typhi***
COL 50 μg	S	COL 50 μg	S
MEM 10 μg	S	MEM 10 μg	S
TIC 75 μg	R	TIC 75 μg	S
AMI 30 μg	S	AMI 30 μg	S

S: Sensitive; I: Intermediate; A: Resistant; ATB: Antibiotics; TIC: Ticarcillin; CEF: Ceftazidime; VAN: Vancomycin; TET: Tetracycline; CEF: Cefalexin; COL: Colistin; AMI: Amikacin.

**Table 4:** Diameter of the inhibition zone of *C. ladanifer* essential oil against the four pathogenic strains.

	*S. aureus*	*E. coli*	*A. baumannii*	*S. Typhi*
*C. ladanifer* 5 μL (EO)	55 ± 0.22	42 ± 0.11	35 ± 0.27	30 ± 0.25

The inhibition diameters were expressed in millimeters (mm): Mean ± SD.

**Table 5:** MIC and MBC of *C. ladanifer* essential oil against selected bacterial strains.

Bacterial Strains		Concentrations (μL/mL)							DMSO 2 μL/mL
		2	10	20	30	40	50	MBC/MIC	
*S. aureus*	MIC	+	−	−	−	−	−	1	+
	MBC	+	−	−	−	−	−		+
*Acinetobacter baumannii*	MIC	+	−	−	−	−	−	1	+
	MBC	+	−	−	−	−	−		+
*E. coli*	MIC	+	−	−	−	−	−	1	+
	MBC	+	−	−	−	−	−		+
*Salmonella Typhi*	MIC	+	−	−	−	−	−	1	+
	MBC	+	−	−	−	−	−		+

+: Presence; −: Absence.

**Table 6:** Values of the inhibition diameters zone of the essential oil of *C. ladanifer*.

Fungal Strains	Growth Inhibition Diameter (GID) (mm)			Fluconazole GID (mm)
	5 μL	10 μL	15 μL	150 mg
*C. albicans*	7	8	10	24.7
*C. tropicalis*	9	11	13	25
*C. glabrata*	9	11	11	23.7
*C. dubliniensis*	8	10	11	19.3
*Candida* sp.	7	8	10	24
*R. rubra*	8	10	12	18.7
*A. niger*	-	7	8	13.7
*C. neoformans*	9	11	13	29.3
*Penicillium* sp.	7	10	12	13.8
*Fusarium* sp.	-	7	8	16.3

**Table 7:** The effect of the *C. ladanifer* essential oil on the MIC and MFC of the selected fungi strains.

	Fungal Strains		
	MIC	MFC	MFC/MIC
*Candida albicans*	32	32	1
*Candida tropicalis*	64	64	1
*Candida glabrata*	32	32	1
*Candida dubliniensis*	32	32	1
*Candida* sp.	16	62	4
*Rhodotorula rubra*	32	32	1
*Cryptoccocus neoformans*	64	64	1
*Penicillium* sp.	64	64	1
*Fusarium* sp.	64	64	1
*Aspergillus niger*	32	32	1

**Table 8:** List of microbial strains tested.

Microbial Strains		
Bacterial Strains	Fungal Strains	
	Yeasts	Molds
*Staphylococcus aureus*	*Candida tropicalis*	*Penicillium* sp.
*Salmonella Typhi*	*Candida glabrata*	*Fusarium* sp.
*Escherichia coli*	*Candida dubliniensis*	*Aspergillus niger*
*Acinetobacter baumannii*	*Candida* sp.	
	*Rhodotorula rubra*	
	*Cryptoccocus neoformans*	

## Data Availability

Data are available upon request.
